# Primary septic arthritis in the elderly – a forgotten killer? Five-year survival and risk factors in a retrospective cohort study

**DOI:** 10.1007/s00068-026-03124-5

**Published:** 2026-02-23

**Authors:** Lars Taubert, Jonas Neijhoft, Christoph G. Wölfl

**Affiliations:** 1 Department for Traumatology and Orthopedics, Goethe University, Theodor-Stern-Kai 7, 60528 Frankfurt am Main, Germany; 2 Department of Orthopaedics, Trauma Surgery and Sports Traumatology, Marienhausklinikum Neuwied-Waldbreitbach, Neuwied, Germany

**Keywords:** Septic arthritis, Joint, Elderly, Infection, Risk factors

## Abstract

**Introduction:**

Primary septic arthritis is an acute emergency associated with high morbidity and mortality in older adults. Rapid diagnosis and treatment are crucial. The study evaluates 5-year survival and identifies prognostic risk factors in elderly patients with primary septic arthritis, including age, American Society of Anesthesiology Physical Status (ASA PS) classification, Charlson Comorbidity Index (CCI), joint involvement, implants, and pathogen spectrum.

**Methods:**

50 patients aged ≥ 60 years treated for primary septic arthritis at two hospitals between 2008 and 2020 were included. Demographic, clinical, and microbiological data were collected retrospectively. Survival was assessed by Kaplan–Meier analysis, with log-rank tests to compare subgroups.

**Results:**

The mean age was 71.2 years. Overall survival was 76% at 1 year and 54% at 5 years. Survival declined significantly with increasing age (p = 0.004), higher ASA PS classification (p < 0.001), higher CCI (p = 0.006), joint involvement (p = 0.027), and presence of implants (p < 0.001). Diabetes mellitus, osteoarthritis, and synovial culture status showed no significant effect. Mean survival by joint ranged from 3.65 for the knee to 2.41 years for the shoulder. Patients with implants had markedly shorter survival (1.1 years vs. 3.84 years). Pathogens were isolated in 70% of cases, most frequently Staphylococcus aureus (38%).

**Conclusion:**

Primary septic arthritis in older adults remains a life-threatening condition with high early- and mid-term mortality. Survival is strongly determined by age, ASA PS classification, CCI, joint involvement, and presence of implants, while comorbidities and pathogen detection show no prognostic relevance.

## Introduction

Primary septic arthritis is a medical emergency associated with a significantly higher morbidity and mortality [1, 2]. Delayed diagnosis or insufficient treatment can lead to sepsis, multiple organ failure and death. It is defined as an acute bacterial infection of a natural joint space caused by an invasive pathogen, leading to rapid destruction of cartilage and bone if not promptly treated [3].

Despite its low overall incidence, varying between 2–10/100,000 people per year in the general population, the number of cases is rising in the recent years [4–9]. Septic arthritis is characterized by a bimodal age distribution, it most frequently affects two distinct groups: young children and older adults. The median age for septic arthritis is around 59 years [4, 8, 9].

Septic arthritis can be further classified in several ways. The condition is divided into acute, with a symptom onset of less than 3 weeks, and chronic, with a symptom onset exceeding 3 weeks [10]. Septic arthritis is also distinguished as monoarticular or polyarticular; approximately 80–90% of cases are monoarticular, whereas only 10–20% present with polyarticular involvement. Polyarticular septic arthritis typically occurs in patients with multiple predisposing factors, such as immunosuppression, rheumatoid arthritis, or severe sepsis, and it most often involves 2–3 joints simultaneously [7, 11, 12]. Another distinction is between hospital-acquired septic arthritis (HASA), which is defined as the development of symptoms at least one month after hospitalization, within six months of hospital discharge, or following outpatient interventions within the preceding six months, and community-acquired septic arthritis (CASA) [13, 14].

Diagnosis is often delayed, as clinical presentation can mimic other arthritides such as rheumatoid arthritis or gout, and common diagnostic tools such as synovial cultures or Gram stains are time-consuming and sometimes unreliable [2, 4].

Risk factors include age, which is associated with characteristics such as immune senescence– an age-related decline in immune function – increased susceptibility and changes in the barrier function of the skin, thereby increasing the risk of infection [5, 7–9, 15]. Further risk factors are prior joint diseases or surgery, diabetes, skin infections or sores, as well as the presence of medical implants [2, 5, 16].

While any joint may be affected, the knee is most common (45–55%), followed by the shoulder (5–10%) [6, 16, 17].

Bacteria can enter the joint cavity via three main pathways: (i) hematogenous spread during a systemic infection, (ii) direct intra-articular inoculation through injections or surgery, or (iii) contiguous spread of a local soft tissue infection by continuity into the joint [2, 16, 18]. By definition, direct inoculation is an etiologic mechanism exclusive to secondary septic arthritis; it is inherently absent in primary septic arthritis, which is characterized solely by hematogenous seeding or contiguous spread. Once in the joint and if left untreated, the infection will rapidly spread into the joint cavity and adjacent synovial membrane [12, 16]. There the bone and cartilage are attacked and, depending on the pathogen, they are causing irreversible damage leading to joint and bone destruction within 2–3 days [4].

Rapid diagnosis with promptly initiated, empiric antibiotic therapy and surgical relief is essential to lower morbidity and mortality [2, 3]. The principal surgical interventions for the affected joint in septic arthritis are arthroscopy and arthrotomy. Arthroscopy, however, is associated with a lower reinfection rate, a reduced initial inflammatory response, and more rapid resumption of activities of daily living [19, 20].

Unfortunately, despite newer diagnostic and therapeutic options, the mortality rate ranges between 6.5 and 48.2%, depending on the study. In addition, following treatment for septic arthritis, patients frequently regain independence in basic activities of daily living; however, their overall health-related quality of life scores often remain below those observed in the general population. Mobility-related activities, such as walking longer distances and climbing stairs, may continue to be impaired, particularly when weight-bearing joints are involved [19, 21, 22].

This retrospective cohort study aimed therefore to analyze the 5-year survival of older adults with septic arthritis. In addition, further information and influencing factors on the bacterial pathogen spectrum as well as possible risk factors and their influence on survival are to be identified and evaluated.

## Materials and methods

### Study design

This retrospective cohort study includes 50 patients aged 60 or above who underwent surgery for primary septic arthritis at St Elisabeth Hospital, Neuwied, Germany, or St. Josef Hospital, Bendorf, Germany, between January 2008 and March 2020. Clinical records were reviewed retrospectively and follow-up was performed until May 31, 2024. The observation period was terminated either upon completion of the respective five-year interval or upon the patient’s death. The last patient under observation died on May 31, 2024, before completion of the five-year interval.

### Patient collective

During the study period, 162 patients were admitted with suspected primary septic arthritis. After applying exclusion criteria, 50 patients were included in the final analysis. In our patient cohort, only monoarticular infections were observed.

Diagnostic criteria of Newman at al. [17] were used: (A) isolation of a pathogenic organism from the affected joint; (B) isolation of a pathogenic organism from another source, such as blood, in association with a clinically suspicious (reddened, overheated) joint suggestive of sepsis; (C) no isolation of a pathogen, but: histological or radiological evidence of infection, or cloudy joint fluid with prior antibiotic treatment and characteristic clinical features; and (D) postmortem or pathological features consistent with septic arthritis [17]. Clinical features were characterized by acute localized symptoms including arthralgia, effusion, erythema, and restricted mobility, alongside systemic manifestations such as pyrexia, tachycardia, and malaise. Laboratory investigations confirmed the diagnosis through elevated inflammatory markers (WBC, CRP, procalcitonin, and ESR) and a synovial leukocyte count exceeding 20,000 cells/µL. Postmortem features were not used in any patient.

Additionally, radiological imaging identified pathognomonic features, including joint destruction, intra-articular effusions, and periarticular abscesses [3, 7, 23]. Differential diagnoses such as rheumatoid arthritis or gout were excluded [1].

Patients were identified retrospectively using hospital archives and ICD-10 codes for infectious arthropathies (M00–M99, M00–M25, M00–M03).

**Fig. 1:**
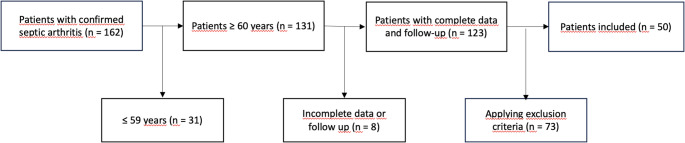
A total of 162 patients with confirmed septic arthritis were initially identified for the study. Of these, 31 patients were excluded as they were younger than 60 years of age. From the remaining 131 patients, 8 were excluded due to incomplete data or loss to follow-up. After further applying specific exclusion criteria to the remaining 123 patients, a final cohort of 50 patients was included in the analysis

The exclusion criteria were as following and are shown in Fig. 1: 31 patients with an age ≤ 59 years were excluded, as well as two patients after a previous (< 6 months) penetrating trauma. 46 patients were excluded with prior (< 6 months) surgery or orthopedic procedure in the area of the affected joint, as well as 6 patients with a periprosthetic or implant-associated joint infection of the affected joint. However, patients with medical implants located in anatomical regions other than the infected joint were included. 19 patients with rheumatoid arthritis, acute gout or crystalloid arthropathy and 8 people with incomplete medical records or insufficient follow-up data were also not included in the study.

### Data collection

A total of 162 cases of infectious arthropathies were collected for the period under review. They were then anonymously analyzed for inclusion or exclusion in our study based on their medical records with regard to our diagnostic criteria for septic arthritis.

The medical records were analyzed for the following points: the reason for admission to the hospital with clinical examination and joint aspiration, which was done in all patients, in the emergency room; blood values, as well as blood cultures, the radiological findings of diagnostic imaging from X-ray, CT or MRI to determine osteoarthritis in the affected joint or to rule out possible osteomyelitis after the first operation; the microbiological results of the samples sent in from the aspirations and joint lavages, as well as the tissue samples obtained intraoperatively and the empirical antibiotic used and, if applicable, its switch to targeted antibiotic therapy after receipt of the microbiological tests.

### Analyzed data

The following patient characteristics were included in the data set for the study: sex, age, side of the affected joint, day of death, medical implant, previous illness, follow-up in days, and ASA PS classification on the respective day of the first operation.

Patient baseline comorbidities were quantified via the Charlson Comorbidity Index (CCI). The CCI is a validated scoring system that quantifies a patient’s comorbidity burden by assigning weighted scores to specific chronic conditions to predict mortality risk [24]. Laboratory markers were analyzed, as well as the results of microbiological findings from joint aspirates and blood cultures.

### Follow-up

For the follow-up, subsequent clinical stays or documents of the respective patients in St Elisabeth Hospital or St Josef Hospital as well as publicly accessible obituaries in newspapers or internet portals and, if permitted by the patient in previous hospital stays, by telephone contact were recorded.

### Statistical analysis

The entire statistical analysis of the data set was performed using SPSS Statistics (Version 29, IBM Corporation, Armonk, USA).

The descriptive analysis of the data was presented with relative and absolute frequencies, standard deviation, median and the arithmetic mean. The correctness of the calculated values was checked using 95% confidence intervals.

The significance of the differences between two or more groups was tested using the t-test. The p-value, which indicates the statistical significance (α-level), was considered statistically significant at *p* < 0.05.

The 1-year and 5-year survival probability of patients with septic arthritis was visualized graphically using Kaplan-Meier curves. In addition, the age groups, the respective classification of the person in the ASA PS classification before the first operation, the presence of medical implants and the affected joint were included in the Kaplan-Meier curves as factors for survival. For each factor, the log-rank test was also used to compare whether the presence of the respective factor had a significant negative impact on patient survival. In addition, the Kaplan-Meier survival curves were used to determine the 5-year mortality in our patient population.

## Results

### Inclusion and pathogen detection

A total of 50 patients were included, comprising 37 men (74%) and 13 women (26%). The mean length of the hospital stay was 25.4 days (± 25 days).

18% (*n* = 9) of cases were categorized as HASA and 82% (*n* = 41) as CASA, between which no significant difference in survival was found.

Comorbidity burden, as measured by the CCI, yielded a mean value of 3.6 (SD ± 2.5).

The knee was the most commonly affected joint (*n* = 33, 66%), followed by the shoulder (*n* = 12, 24%), hip (*n* = 2, 4%), upper ankle (*n* = 2, 4%), and sacroiliac joint (*n* = 1, 2%). The right side was involved more frequently than the left (66% vs. 34%).

All patients underwent arthroscopic surgery. In 11 cases (22%), arthrotomy was used in subsequent surgeries.

Patient age at diagnosis ranged from 60 to 95 years (mean 71.2 years). The mean follow-up was 3.29 years (1200 days; SD ± 108.5 days, range 3–1825 days). Follow-up was complete in 86,8%. Overall survival was 84% at 30 days, 76% at 1 year and 54% at 5 years. Overall, the successful follow-up rate was 100%.

A higher ASA PS classification, higher CCI, increasing age, the affected joint, as well as the presence of medical implants showed a significant impact on 5-year survival (*p* < 0.05). No significant influences were found for diabetes mellitus, osteoarthritis and various pathogens. All demographic characteristics can be seen in Table 1.

**Table 1:** Distribution of demographic factors devided into sex and overall. This table summarizes the demographic findings, predominant illnesses, ASA PS classification and joint location

	Female	Male	Total
Number of patients [*n* (%)]	13 (26)	37 (74)	50 (100)
Age in years [Mean (Range)]	74.8 (60–95)	69.9 (60–89)	71.2 (60–95)
**Affected joint – Knee**			
Right [n (%)]	4 (20)	16 (80)	20 (40)
Left [n (%)]	1 (7.7)	12 (92.3)	13 (26)
**Affected joint – Shoulder**			
Right [n (%)]	4 (40)	6 (60)	10 (20)
Left [n (%)]	2 (100)	0 (0)	2 (4)
**Affected joint – Ankle**			
Right [n (%)]	0 (0)	1 (100)	1 (2)
Left [n (%)]	0 (0)	1 (100)	1 (2)
**Affected joint – Hip**			
Right [n (%)]	1 (100)	0 (0)	1 (2)
Left [n (%)]	1 (100)	0 (0)	1 (2)
**Affected joint – Sacroiliac**			
Right [n (%)]	0 (0)	1 (100)	1 (2)
**ASA PS Classification**			
ASA I [n (%)]	0 (0)	0 (0)	0 (0)
ASA II [n (%)]	0 (0)	2 (100)	2 (4)
ASA III [n (%)]	9 (26)	26 (74)	35 (70)
ASA IV [n (%)]	4 (31)	9 (69)	13 (26)
Positive synovial fluid culture [n (%)]	11 (31)	24 (69)	35 (70)
Medical implants [n (%)]	2 (20)	8 (80)	10 (20)
Osteoarthritis [n (%)]	12 (32)	26 (68)	38 (76)
Diabetes mellitus [n (%)]	2 (14)	12 (86)	14 (28)
Mean CCI	3.4	3.8	3.6

Pathogens were identified in 35 of 50 cases (70%). *Staphylococcus aureus* was most frequent (*n* = 19, 38%), followed by *Escherichia coli* (*n* = 8, 16%). Table 2 lists all microbiological findings.

### 5-year survival by age groups

**Fig. 2:**
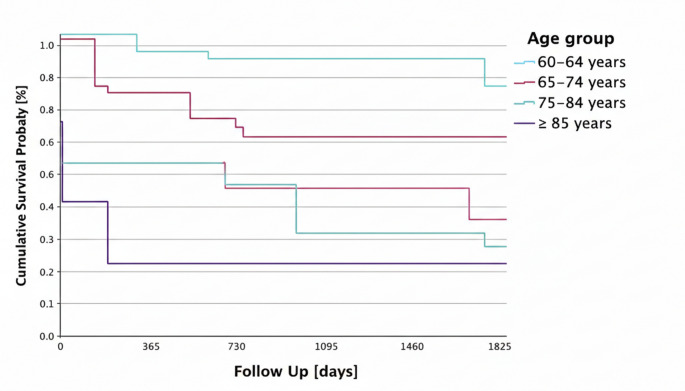
Kaplan-Meier five-year survival by age group. This figure depicts the cumulative five-year survival probabilities stratified by age categories, demonstrating a progressive decline in survival with increasing age (log-rank *p* = 0.004). Notably, patients aged 60–64 years show nearly fourfold longer survival compared to those ≥ 85 years. While younger patients are expected to survive longer than the oldest age group in the general population, the magnitude of the observed survival difference appears substantially larger than expected from physiological ageing alone

A significant difference in 5-year survival was found between the different age groups. The chi-square test for the correlation between 5-year survival and the respective age classification yielded a value of 13.327. The cumulative survival probability of the groups is shown in Fig. 2. The average survival of the entire study population was 3.29 years (SD ± 108.5 days; 95% CI: 987.6–1412.9).

There were 14 (28%) patients in the age group 60–64 years. They had an average follow-up of 4.35 years (SD ± 155 days; 95% CI: 1283.2–1894.6). In the 19 (38%) 65-74-year-old patients, the mean follow-up was 3.54 years (SD ± 169.5 days; 95% CI: 959.2–1623.3). In the 12 (24%) 75-84-year-olds, the mean survival was 2.53 years (SD ± 212.5; 95% CI: 507.3–1340.2). The 3 (6%) patients aged ≥ 85 years showed a mean survival of 1.18 years (SD ± 350.9 days; 95% CI: 0.00–1117.2).

So overall survival decreased progressively with age (log-rank *p* = 0.004). Mean survival ranged from 4.35 years in patients aged 60–64 years to 1.16 years in those ≥ 85 years. The youngest age group thus had nearly fourfold longer survival compared to the oldest.

### 5-year survival by ASA PS classification

**Fig. 3:**
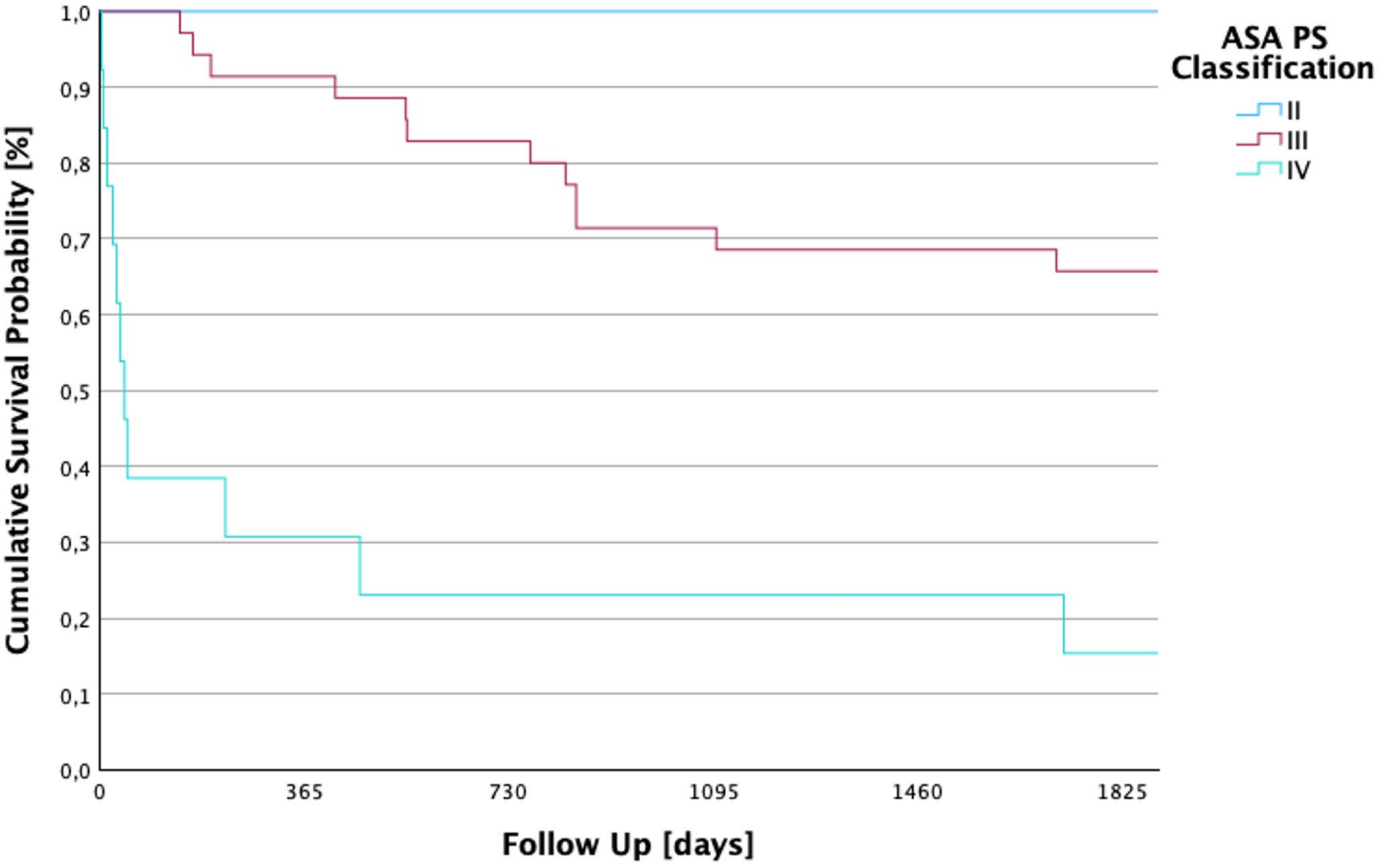
Kaplan–Meier five-year survival by ASA Physical Status classification. This figure shows the cumulative 5-year survival curves separated by ASA PS class (log-rank *p* < 0.001), demonstrating a significant reduction in survival with increasing ASA severity. Patients in ASA II display full long-term survival, whereas ASA IV patients experience a dramatic reduction to ~ 1.3 years, illustrating the prognostic value of perioperative physiological status

A higher ASA PS classification was significantly associated with poorer 5-year survival (log-rank *p* < 0.001). Patients classified as ASA II survived up to 5 years, while mean survival declined to 3.92 years in ASA III and 1.32 years in ASA IV patients as seen in Fig. 3. The chi-square test for the correlation between 5-year survival and the respective ASA PS classification yielded a value of 23.499. The cumulative survival probability of the different ASA PS classifications is shown in Fig. 3.

A total of 2 (4%) patients were classified with ASA II. These had an average follow-up of 5 years (SD ± 1825 days; 95% CI: 1825–1825). 35 (70%) patients were classified as ASA PS III. In this group, the mean follow-up was 3.92 years (SD ± 102.9 days; 95% CI: 1229.8–1633.1). 13 (26%) patients were also assigned to the ASA PS IV group. There, the mean value of the follow up was 1.32 years (SD ± 209.4 days; 95% CI: 70.2–893.2).

### 5-year survival by Charlson comorbidity index

**Fig. 4:**
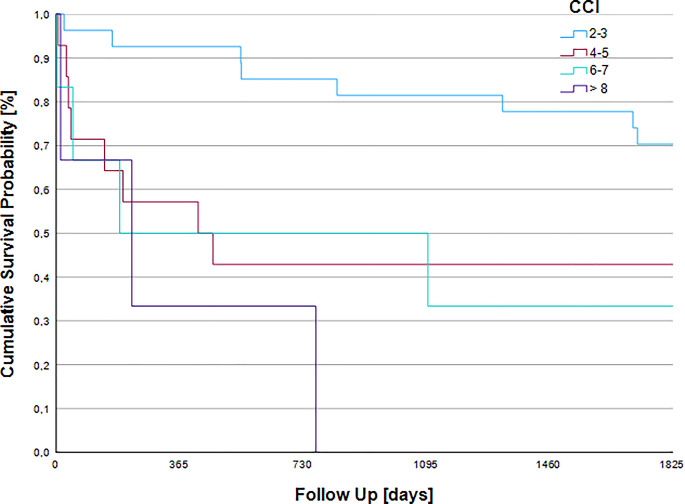
Kaplan-Meier five-year survival by CCI. This figure depicts the cumulative five-year survival probabilities stratified by different CCI scores, demonstrating a progressive decline in survival with increasing CCI score (log-rank *p* = 0.006). Notably, patients with a CCI score of ≥ 8 have fourfold lower survival compared to patients with a CCI of 2–3. It is noteworthy that the mean survival duration remained relatively comparable between the CCI 4–5 and 6–7 cohorts, differing by a mere 50 days. This suggests that within these intermediate-to-high comorbidity brackets, the CCI score may have limited incremental prognostic value for survival duration

A significant difference in 5-year survival was found between the different CCI (log rank *p* = 0.006). The chi-square test for the correlation between 5-year survival and the respective CCI yielded a value of 12.544. Patients classified as CCI 2–3 survived up to 4.22 years, while mean survival declined to 2.41 years in CCI 4–5, 2.28 years in CCI 6–7 and 0.92 years in CCI ≥ 8 patients as seen in Fig. 4. The cumulative survival probability of the groups is shown in Fig. 4.

The distribution of comorbidity severity, as measured by the CCI, revealed that the majority of the cohort (*n* = 27, 54%) presented with a CCI score of 2–3. A further 14 patients (28%) had a score of 4–5, while 6 patients (12%) were categorized with a score of 6–7. Only a small minority (*n* = 3, 6%) exhibited a high comorbidity burden with a CCI ≥ 8.

### 5-year survival by affected joint

**Fig. 5:**
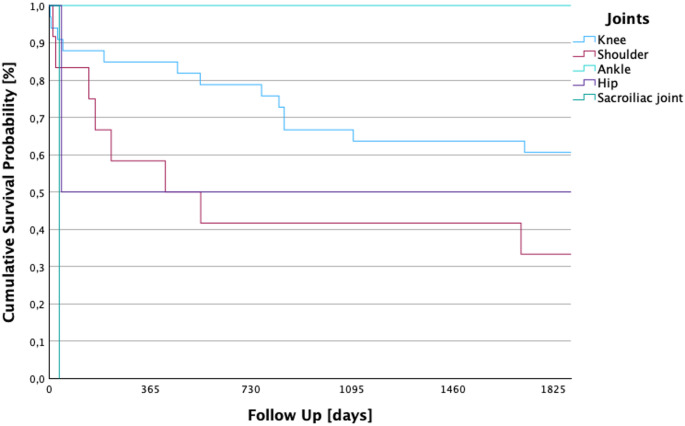
Kaplan–Meier five-year survival according to the affected joint region. This figure depicts survival depending on the infected joint (*p* = 0.027), with the ankle joint showing the best prognosis and the sacroiliac joint the worst. These findings imply that anatomical location and joint-specific surgical accessibility may influence infection clearance and long-term outcomes

Survival varied by affected joint (*p* = 0.027). Mean survival was longest in ankle infections (5 years) and shortest in the sacroiliac joint (0.1 years). Knee infections showed intermediate survival (3.65 years), followed by hip (2.56 years) and shoulder (2.41 years) involvement as seen in Fig. 5: The chi-square test for the correlation between 5-year survival and the respective affected joint yielded a value of 10.984.

In a total of 33 (66%) patients, the knee was the affected joint in primary septic arthritis. The mean survival of these patients was 3.65 years (SD ± 121.6 days; 95% CI: 1092.4–1569.2). The group of patients with septic arthritis of the shoulder included 12 (24%) patients. They had an average follow-up of 2.41 years (SD ± 239.1 days; 95% CI: 410–1347.3). In the 2 (4%) patients with an affected upper ankle joint, the mean follow-up was 5 years (SD ± 1825 days; 95% CI: 1825–1825). In the 2 (24%) patients with septic arthritis of the hip, the mean survival was 2.56 years, (SD ± 890.5; 95% CI: 0.00–2679.9). The 1 (2%) patient in whom the sacroiliac joint was affected showed a mean survival of 0.1 years (SD ± 0 days; 95% CI: 36).

### 5-year survival by medical implants

**Fig. 6:**
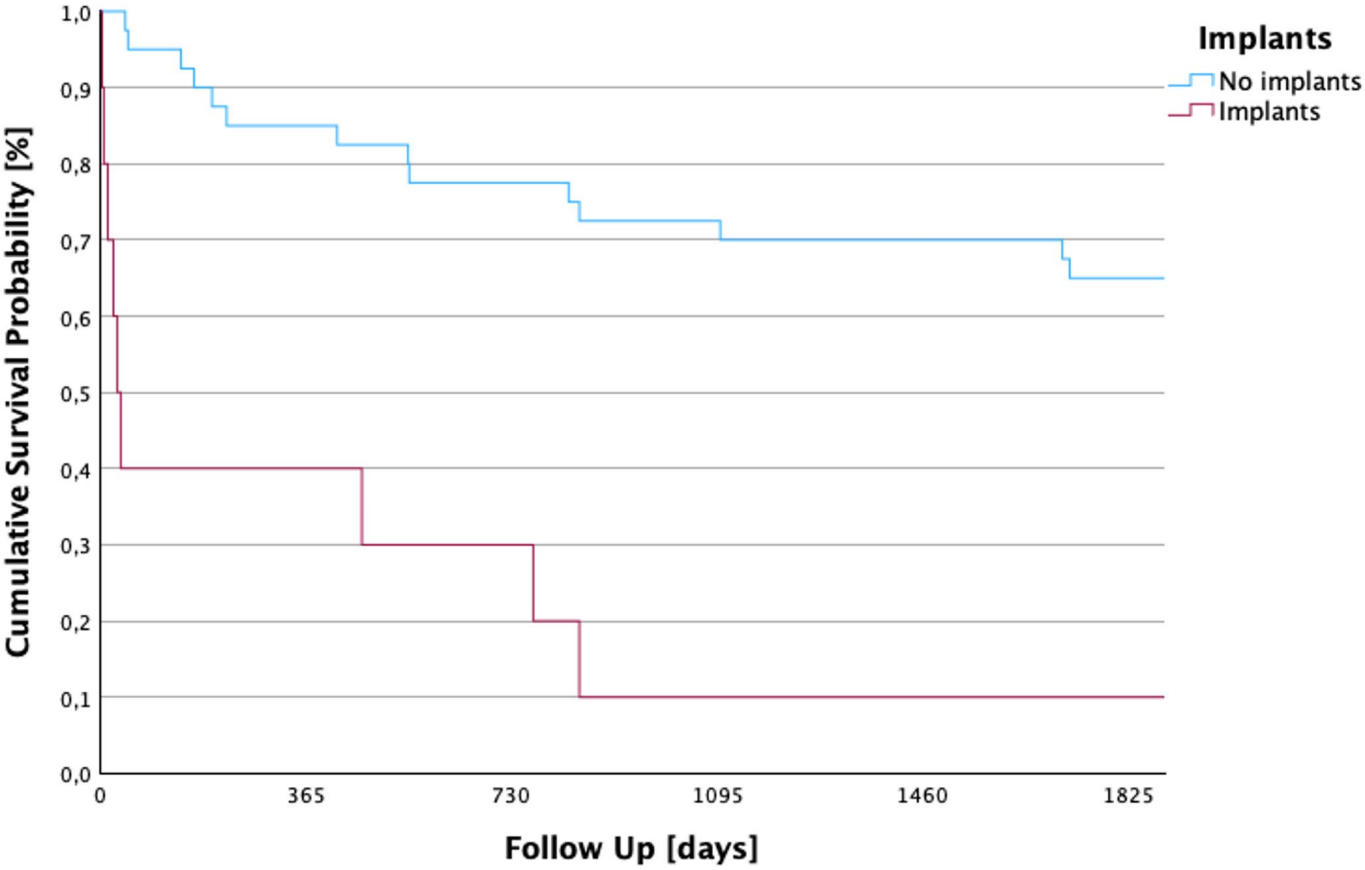
Kaplan–Meier five-year survival by presence of medical implants. This figure demonstrates a reduction in long-term survival among patients with pre-existing implants at different location than the affected joint compared to those without (*p* < 0.001). The steep early mortality drop among implant carriers suggests the persistent biofilm-mediated infection and systemic vulnerability associated with the presence of foreign material. The findings of this study indicate that the risk associated with septic arthritis extends beyond periprosthetic or peri-implant infections; notably, the presence of an unrelated medical implant at any anatomical site is concurrently associated with a significantly poorer prognosis

A significant difference in 5-year survival was found between patients with a medical implant and patients without (log rank *p* < 0.001, see Fig. 6) Patients with a predominant periprosthetic infection of the affected joint were excluded from this study. Patients with medical implants located in anatomical regions other than the affected joint were included. The chi-square test for the correlation between 5-year survival and patients with and without medical implants yielded a value of 22.499.

A total of 40 (80%) patients had no medical implants at presentation with primary septic arthritis. They had an average follow-up of 3.84 years (SD ± 106.3 days; 95% CI: 1191.6–1608.2). In the total of 10 (20%) patients with a medical implant of any kind, which was not localized in the affected joint, the average follow-up was 1.1 years (SD ± 189.3 days; 95% CI 29.5–774.1).

### Microbial spectrum and synovial cultures

In 15 cases (30%), no successful detection of bacteria was possible. However, in 35 out of 50 patients, a positive detection of bacteria was achieved (70%). Of these, 25 cases (71.4%) showed Gram-positive bacteria. In 10 cases (28.6%), Gram-negative bacteria were detected. A total of six different pathogens were found. The most frequently detected pathogen was *Staphylococcus aureus* with 19 detections (38%), of which 4 cases (8%) were *MRSA*. The next most common pathogen was *Escherichia coli* with 8 detections (16%). The results can be seen in Table 2.

**Table 2:** Distribution of pathogens isolated from synovial samples. This table summarizes the Microbiological findings, dividing detected pathogens into Gram-positive and Gram-negative groups, including frequency of MRSA strains. Staphylococcus aureus is highlighted as the predominant organism (38%), reflecting its well-established role as a dominant agent in septic arthritis. The relatively high rate of culture-negative cases (30%) emphasizes the limitations of pathogen detection in diagnosis

Pathogen	*n*
**Negative synovial fluid culture**	15 (30%)
**Grampositive**	
*Staphylococcus aureus [MRSA]*	19 (38%) [4 (8%)]
*Staphylococcus epidermidis*	4 (8%)
*Streptococcus parasanguinis*	1 (2%)
*Staphylococcus hominis*	1 (2%)
**Total Grampositive**	25 (50%)
**Gramnegative**	
*Escherichia coli*	8 (16%)
*Pseudomonas aeruginosa*	2 (4%)
**Total Gramnegative**	10 (20%)
**Total**	50 (100%)

**Fig. 7:**
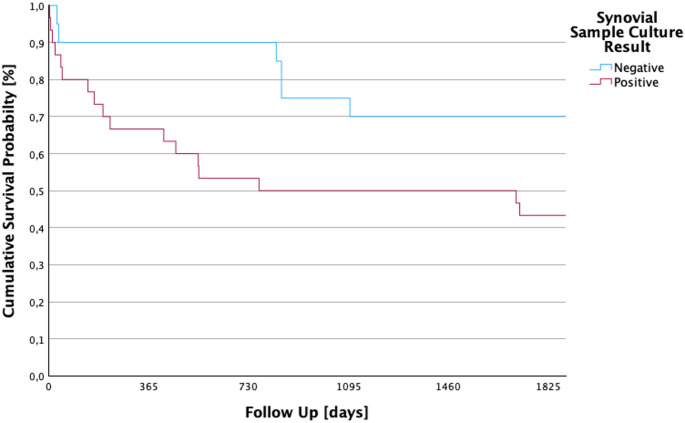
Kaplan–Meier five-year survival by synovial culture status. This graph compares survival rates of culture-positive and culture-negative cases, showing no statistically significant difference (*p* = 0.089). Notably, culture-negative patients even show a numerically longer survival, supporting the hypothesis that clinical severity rather than identification of the pathogen may drive mortality risk

No significant difference in 5-year survival was found between patients with positive and negative synovial cultures (*p* = 0.089, see Fig. 7). The chi-square test for the correlation between 5-year survival and a positive or negative synovial culture result yielded a value of 2.887. A total of 15 (30%) patients had no pathogen detection in the synovial cultures. They had an average follow-up of 3.99 years (SD ± 173.3 days; 95% CI: 1115.2–1794.4).

In 35 (70%) patients, one or more pathogens were detected in the synovial culture. In this group, the average follow-up was 2.99 years (SD ± 133.3 days; 95% CI: 830–1352.5).

## Discussion

This retrospective study evaluated medium-term survival in 50 patients with primary septic arthritis over a 12-year period. The mean survival was 3.29 years, with a 1-year and 5-year survival rate of 76% and 54%, respectively.

Age, ASA PS classification, joint location, and presence of implants were identified as significant prognostic factors, whereas osteoarthritis, diabetes, and gram stain results showed no significant association with survival.

Gram-positive bacteria were the predominant pathogens (50% vs. 22% Gram-negative), with *Staphylococcus aureus* being the most frequently isolated organism. In 30% of cases, no pathogen could be identified. *Staphylococcus aureus* was by far the most common pathogen (38%), followed by *E. coli* (16%).

### Survival rate comparison

In the present study, the mean survival decreased progressively with advancing age, recorded at 4.35 years for patients aged 60–64, 3.54 years for those aged 65–74, 2.53 years for those aged 75–84, and 1.18 years for those aged 85 years and older. The overall mean survival for the entire cohort was 3.29 years. In contrast, the age-matched life expectancy for the general population in Germany is substantially higher, reported at 21.8/25.4 years (male/female), 14.4/17.0 years, 8.1/9.6 years, and 3.7/4.3 years for the respective age brackets [25]. This underscores a profound reduction in longevity among patients with septic arthritis, whose average life expectancy is approximately fourfold shorter across nearly all age groups.

Mortality rates are also markedly increased compared to other orthopedic injuries, with some estimates demonstrating a twofold increase. Specifically, the reported one-year mortality rate for periprosthetic fractures is 17.7%, exceeding the rates observed for hip fractures (16.6%) and distal femur fractures (13.4%) [26–28].

When compared with national life expectancy and other orthopedic conditions such as hip or periprosthetic fractures, overall survival in patients with septic arthritis—particularly of the shoulder—was markedly reduced [26, 27]. To contextualize the mortality associated with primary septic arthritis, five-year mortality rates were compared with those reported for major malignancies in recent population-based analyses. The overall five-year mortality rate for patients with breast cancer is 16.3% [29], 10% for those with prostate cancer [30], and 31.7% for patients with colorectal cancer [31]. In comparison with these findings, our results demonstrate substantially higher mortality rates, with one-/five-year mortality for primary septic arthritis of 24%/46% on average whereas 5-year mortality rises to 66.7% in the case of an affected shoulder joint.

These findings are consistent with those of comparable studies focusing exclusively on geriatric patients with primary septic arthritis. These studies report one-year mortality rates ranging from 23.3% to 35.7%, and five-year mortality rates ranging from 52.6% to 64.3% [32, 33]. Broader epidemiological studies that encompass all age groups and clinical subtypes of septic arthritis demonstrate a mortality of 11% − 23% [13, 21, 22].

The higher mortality in patients with an infected shoulder joint may be attributable to the anatomical separation of the shoulder joint into the glenohumeral joint and the subacromial space, which are divided by the rotator cuff. Infection may potentially spread between these two compartments, complicating surgical eradication. Furthermore, the clinical manifestations of septic arthritis in the shoulder may present later than in other joints, potentially delaying both surgical intervention and antibiotic therapy, which may contribute to poorer outcomes.

The study underscores that septic arthritis can reflect an underlying compromised immune system and highlights the critical importance of early diagnosis and management, particularly in patients with systemic risk factors or joint implants. Notably, the patient presenting with an affected sacroiliac joint only survived 36 days. However, due to the very small subgroup size of only one patient, the result is statistically limited and does not allow for broader statistical generalization.

A strong association between ASA PS Classification and 5-year survival was observed. Higher ASA PS classifications were associated with increased risk of postoperative complications, mortality, and wound infections, being consistent with previous literature [34, 35]. This reflects the fact that patients with ASA IV often have severe systemic diseases and typically undergo surgery only in emergencies, such as septic arthritis, making outcomes worse. As a result, the ASA PS classification of physical condition has been described as a valuable prognostic variable for postoperative medical complications and mortality [34, 35]. Our study aligns with these findings. A higher ASA-PS classification is associated with a higher mortality rate, as these patients suffer from diseases that pose a permanent threat to life even without septic arthritis.

This is consistent with our findings, which demonstrate that a higher CCI is significantly associated with increased mortality in patients with septic arthritis. The findings underscore a critical correlation between pre-existing health status (preoperative ASA PS Score and CCI and survival outcomes in the context of acute joint infections. Specifically, patients presenting with a higher CCI demonstrated a markedly increased susceptibility to adverse clinical outcomes [13, 36]. This observation is rooted in the fact that an elevated CCI serves as a quantifiable proxy for multimorbidity and systemic frailty [36, 37].

From a pathophysiological perspective, these individuals possess a diminished physiological reserve, which significantly compromises their ability to mount an effective immunological response to the septic insult [7, 38]. The systemic complexity inherent in high-CCI cohorts - often involving a synergistic interplay between cardiovascular, metabolic, and renal pathologies -not only complicates the surgical and pharmacological management of the infection but also serves as a robust predictor of all-cause mortality.

Consequently, these findings indicate that in the geriatric population the severity of the underlying comorbidity burden is at least as decisive for long-term prognosis as pathogen virulence or the timing of surgical intervention.

### Joint location

The knee was the most frequently affected joint, aligning with previous studies [16, 23, 39]. This may reflect the higher prevalence of degenerative joint disease in the knee compared to other joints, as osteoarthritis is an important risk factor for the development of septic arthritis [2, 15]. The association between osteoarthritis and septic arthritis supports the concept that joint degeneration in the elderly increases vulnerability to infection.

### Pathogen influence and medical implants

*Staphylococcus aureus* is the most common and significant pathogen. It should be noted that not only *Staphylococcus aureus*, but also other bacteria such as *Staphylococcus epidermidis* or *Pseudomonas aeruginosa* form biofilms on implants.

Patients with medical implants anywhere in the body but the affected joint had markedly reduced survival, with 1-year and 5-year mortality rates of 60% and 90%, respectively, compared to 15% and 35% in patients without implants. Medical implants are a known risk factor for infections, including septic arthritis, largely due to biofilm formation by above-mentioned bacteria [40, 41]. Biofilms, which develop in stages on implant surfaces, protect bacteria and enable persistent infections with potential for systemic spread [42]. Moreover, implant carriers often present with multiple comorbidities, further increasing susceptibility and mortality risk.

### Diabetes mellitus

In this study, diabetes mellitus did not have a statistically significant impact on survival in patients with primary septic arthritis, with one- and five-year mortality rates of 35.7% and 64.3%, respectively, compared with 25% and 41.7% in non-diabetic patients. Although not significant, these data suggest higher mortality in patients with diabetes, consistent with previous reports linking diabetes to increased susceptibility to bacterial infections and infection-related mortality [43, 44]. Mechanisms such as hyperglycemia, immune dysfunction, and increased risk of infection with resistant strains may enhance bacterial growth and virulence, including biofilm formation and tissue adhesion [40, 45, 46]. Accordingly, pre-existing diabetes mellitus may increase both the risk and severity of septic arthritis, contributing to poorer outcomes in affected patients.

### Limitations and strengths

Retrospective studies, such as this one, face several limitations including data loss, unrecorded variables, and inconsistent documentation, which can affect the reliability of statistical results [47].

Furthermore, telephone calls and obituaries were utilized to ascertain mortality status. This approach may introduce selection bias, as deaths are more likely to be recorded among individuals with public or social visibility, potentially leading to under-ascertainment of mortality among marginalized populations, non-local residents, or individuals lacking family networks to report deaths. Telephone follow-up is additionally susceptible to non-response bias, because healthier survivors are more likely to participate, whereas frailer patients or those with post-infectious cognitive decline are systematically underrepresented.

Furthermore, misclassification bias may occur. Obituaries and telephone reports rely primarily on proxy information from relatives rather than on medical records, increasing the risk of inaccurate attribution of death to septic arthritis versus underlying comorbidities. The absence of detailed clinical data in obituaries can therefore lead to both underestimation and overestimation of septic arthritis as the underlying cause of death.

Over the 12-year study period, changes in diagnostics and treatment approaches introduced potential performance bias. It should be noted that almost all patients only underwent surgery once. Current guidelines and literature recommend repeated operations until sufficient reduction in laboratory inflammation values and clinical findings is achieved.

Furthermore, specific clinical variables including body mass index (BMI), intensive care unit (ICU) length of stay, and primary infection foci were not systematically documented, limiting our ability to assess their prognostic significance. Additionally, a follow-up rate of 86.8% may have led to selection bias and skewed survival data, especially for younger patients. While retrospective studies cannot establish causality, they are useful for analyzing rare diseases over long periods and identifying potential risk factors with relatively stable follow-up rates.

### Future aspects

In the context of demographic change and an increasingly ageing population, the incidence of primary septic arthritis is expected to rise. Given the persistently high mortality associated with this condition, further research is urgently needed. Future studies should prioritize well-designed, prospective, multicenter trials with sufficient case numbers to generate robust and generalizable data. These studies should evaluate empiric versus targeted antibiotic regimens in light of evolving treatment guidelines, pathogen prevalence, and resistance patterns. Moreover, further improvement and implementation of standardized diagnostic and therapeutic guidelines are essential to optimize patient outcomes and reduce the considerable morbidity and mortality associated with this disease.

## Conclusion

Primary septic arthritis in the elderly is far from a resolved problem—it remains a devastating and frequently fatal condition. Despite advances in diagnostics and therapy, almost half of the affected patients die within five years of diagnosis. Survival is strongly determined by preoperative ASA PS classification, age, CCI, the affected joint, and the presence of implants, whereas common comorbidities such as diabetes or osteoarthritis appear to have limited impact.

The high proportion of culture-negative cases emphasizes that early surgical intervention and empiric antibiotic coverage remain the cornerstones of the treatment.

With *Staphylococcus aureus* as the dominant pathogen, empiric regimens should continue to target Gram-positive organisms. Future prospective multicenter studies are urgently needed to develop and improve diagnostic and therapeutic pathways to improve survival in this vulnerable and growing population.

## Data Availability

The data originate from a surgical and traumatological care unit at St. Elisabeth Hospital, Neuwied, as well as St. Josef Hospital, Bendorf, The data can be provided anonymously in a separate file upon request. A public dataset was not used to obtain the data presented.

## Abbreviations

ASA PS Classification: American Society of Anesthesiology Physical Status Classification
BMI: Body mass index.
CASA: Communtiy acquired septic arthritis
CCI: Charlson Comorbidity Index
CRP: C-reactive protein
E. coli: Escherichia coli
ESR: Erythrocyte sedimentation rate
HASA: Hospital acquired septic arthritis
ICU: Intensive care unit
CI: Confidence interval
SD: Standard deviation
WBC: White blood cel
